# An ecosystem-wide reproductive failure with more snow in the Arctic

**DOI:** 10.1371/journal.pbio.3000392

**Published:** 2019-10-15

**Authors:** Niels Martin Schmidt, Jeroen Reneerkens, Jens Hesselbjerg Christensen, Martin Olesen, Tomas Roslin

**Affiliations:** 1 Department of Bioscience, Aarhus University, Roskilde, Denmark; 2 Arctic Research Centre, Aarhus University, Aarhus, Denmark; 3 Groningen Institute for Evolutionary Life Sciences (GELIFES), University of Groningen, Groningen, The Netherlands; 4 NIOZ Royal Netherlands Institute for Sea Research, Department of Coastal Systems and Utrecht University, Den Burg, Texel, The Netherlands; 5 Niels Bohr Institute, University of Copenhagen, Copenhagen, Denmark; 6 Danish Meteorological Institute, Copenhagen, Denmark; 7 NORCE climate, Bergen, Norway; 8 Department of Ecology, Swedish University of Agricultural Sciences, Uppsala, Sweden; 9 Department of Agricultural Sciences, University of Helsinki, Helsinki, Finland

## Abstract

2018: Arctic researchers have just witnessed another extreme summer—but in a new sense of the word. Although public interest has long been focused on general warming trends and trends towards a lower sea ice cover in the Arctic Ocean, this summer saw the realization of another predicted trend: that of increasing precipitation during the winter months and of increased year-to-year variability. In a well-studied ecosystem in Northeast Greenland, this resulted in the most complete reproductive failure encountered in the terrestrial ecosystem during more than two decades of monitoring: only a few animals and plants were able to reproduce because of abundant and late melting snow. These observations, we suggest, should open our eyes to potentially drastic consequences of predicted changes in both the mean and the variability of arctic climate.

## Abstract 2

In summer 2018, many arctic regions experienced unprecedented large amounts of snow. This Perspective article reports the ecological impacts of this extreme event, and shows that the severe snow conditions resulted in an almost complete reproductive failure across the entire ecosystem. The 2018 may be a rare event, but it also offers a gloomy peep into the future.

## Climate change is not just “warming”

Around the world, temperatures are increasing. To predict the consequences for local ecosystems, we typically rely on the assumption that we can predict future conditions from current trends, and that with climate change, species and communities will follow their climatic envelopes in an orderly manner [1]. Yet, at least three considerations complicate this paradigm: First, current predictions involve changes in both the mean and the variance of climatic parameters [2–4]. Here, changes in the variance may have as important ecological consequences as changes in the mean. Second, although scenario analyses tend to focus on predictions regarding temperature, current predictions of climate change posit that many other parameters will change, too—most notably precipitation in many parts of the world [4,5]. Of arctic ecosystems, many are limited by snow conditions and water availability [6], and changes in precipitation may prove as crucial as changes in temperature—if not even more. Third, many current predictions are based on the idea of gradual, directional change, thus allowing for simple extrapolation of ecological effects (e.g., [7]). However, recent history includes many examples of so called tipping points [8–10], in which a relatively subtle change in conditions abruptly flips the system from one state to another. Such shifts may be hard to foresee, and their consequences impossible to assess from past experience.

## The arctic extreme of 2018

**Fig 1:**
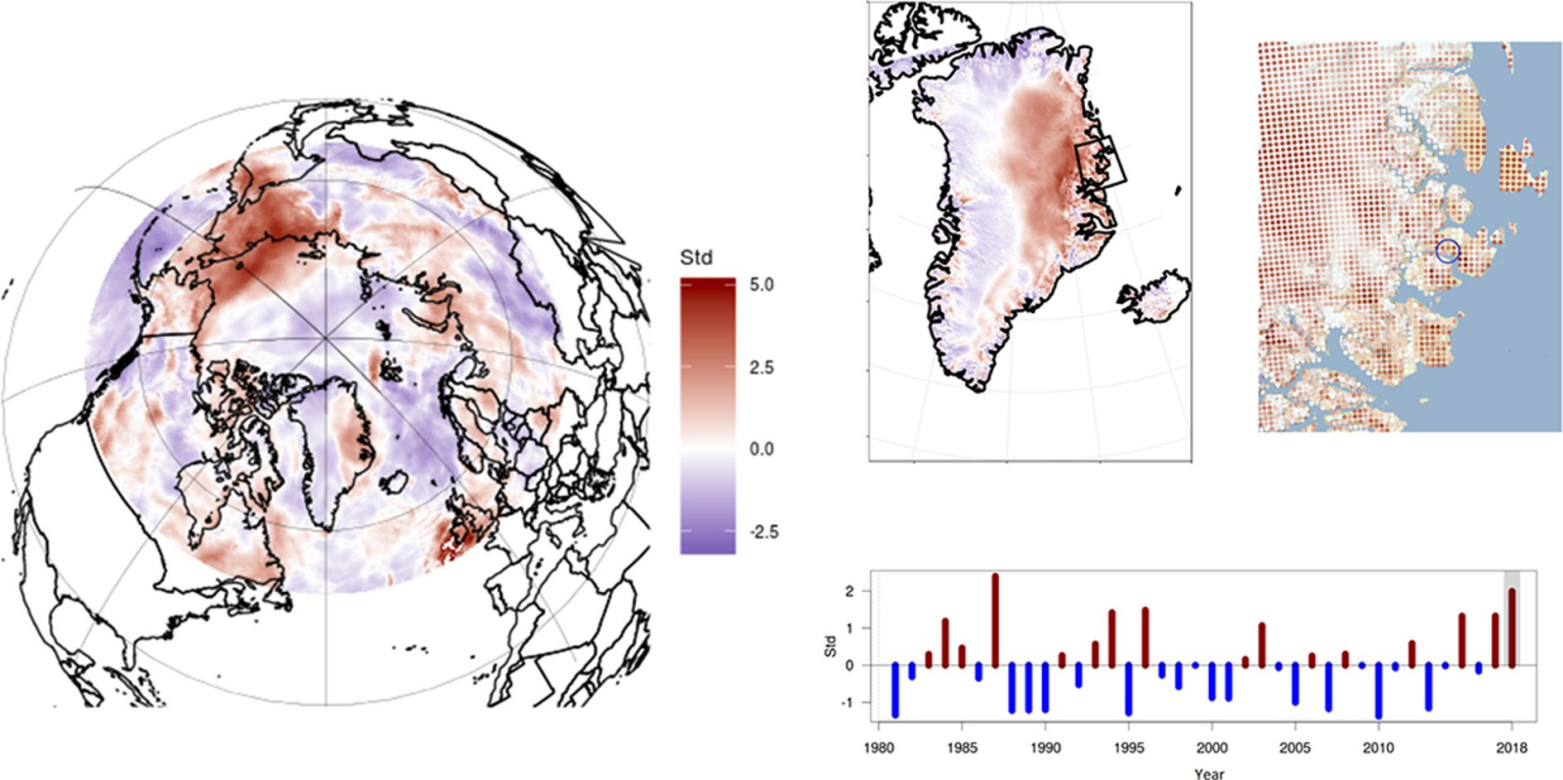
The 2018 snow cover compared with long-term precipitation patterns in the Arctic. The three maps show the deviation (in units of standard deviations) of the 2018 season from the 1980 to 2018 normalized precipitation curve on the Pan-Arctic scale (left), the Greenland scale (top middle), and the Zackenberg region (top right), with the black circle marking Zackenberg. For each year, snow precipitation was calculated as total precipitation in November through April, obtained from a high-resolution data-assimilation system and a regional climate model (see Supporting information). The lower right panel shows the annual deviation (in units of standard deviations) from the normalized precipitation curve in the Zackenberg drainage basin from 1980 to 2018. Additional information about data and analyses can be found in S1 Data collection and analyses. Data presented here are available at http://prudence.dmi.dk/data/temp/MOL/PLOS.

The summer of 2018 underscored all three concerns. Beyond the general trend of warmer and earlier summers and a retreating snow cover [11], large parts of the Arctic, and in particular, the High Arctic, were covered by unusually large amounts of snow in 2018 (Fig 1). This pattern was particularly evident in Northeast Greenland (Fig 1) and at the research station of Zackenberg (Fig 1), where the local snow precipitation deviated from long-term mean conditions by several standard deviations. At Zackenberg, this resulted in snow melt being extraordinarily delayed.

The conditions of 2018 obviously comprise only a single annual data point along a long-term, noisy trajectory. Thus, we do not want to overinterpret a single observation in one location but merely to relate the events of 2018 to a long-term, ecosystem-level perspective—pointing to what more years with more extreme snow conditions may cause, not least if they increase in frequency. Here, monitoring sites, such as Zackenberg in Northeast Greenland [12], offer crucial time series of biota over many years. Where robust, mechanistic climate predictions can be made based on the law of physics, predictions of ecological consequences of altered climatic conditions are likely to be far more inaccurate [13]. Thus, ecosystem-level monitoring sites allow us to examine trends and variability and to quantify the ecological consequences of extreme events, such as the 2018 snow situation.

## Consequence: An ecosystem-wide reproductive collapse

**Fig 2:**
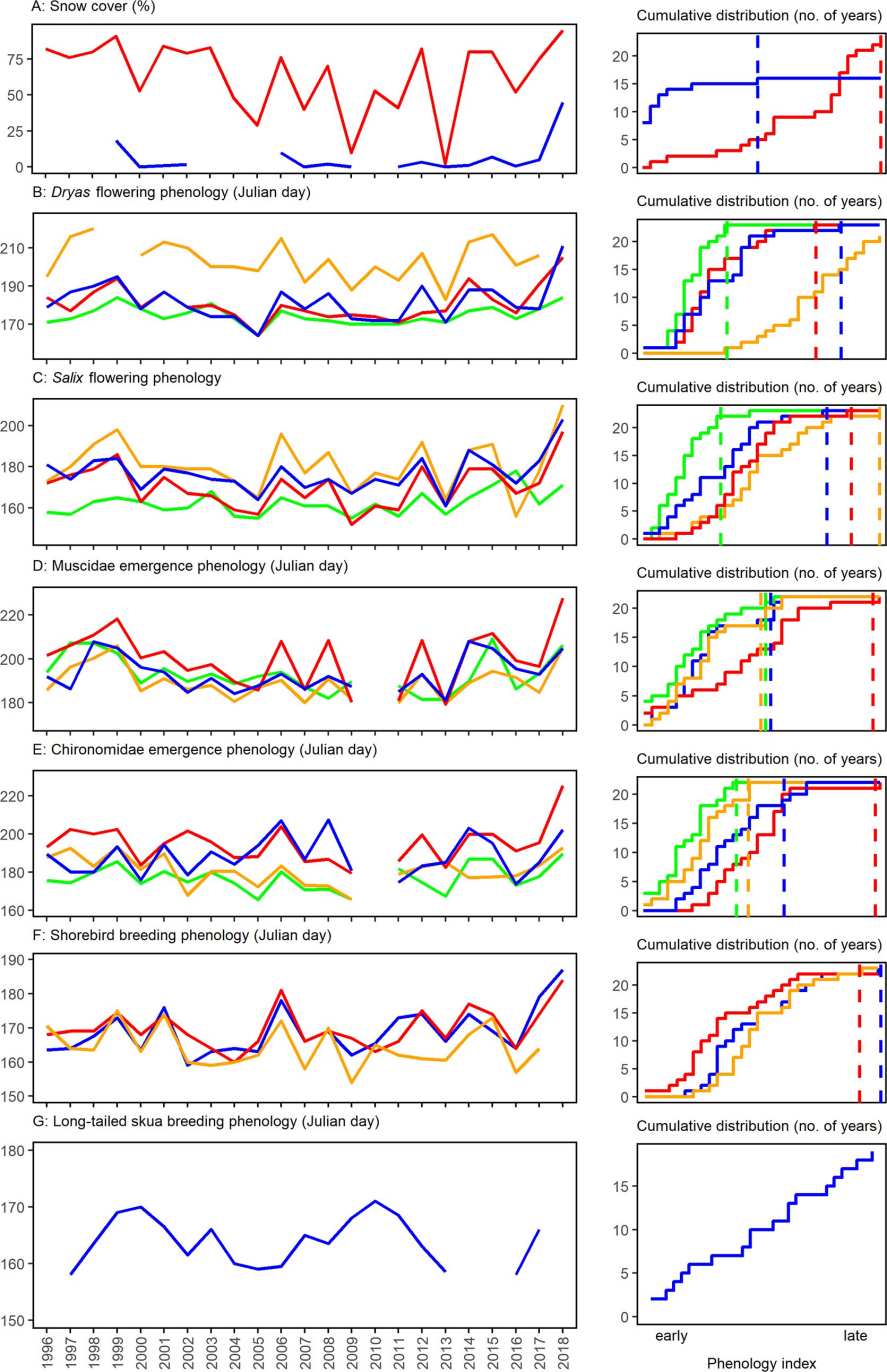
Ecological time series on phenology from Zackenberg in Northeast Greenland. Individual panels show records on abiotic conditions and phenological events and their associated cumulative distribution plots: (A) Snow cover in second week of June (red) and in third week of July (blue), (B) Julian date for 50% flowering of *Dryas* sp. in four permanent plots, (C) Julian date for 50% flowering of *Salix arctica* in four permanent plots, (D) Julian date for 50% flowering of Muscidae emergence in four permanent plots, (E) Julian date for 50% flowering of Chironomidae emergence in four permanent plots, (F) median date for nest initiation of three shorebird species most common in Zackenberg (blue: Dunlin *Calidris alpina*; red: Sanderling *C*. *alba*; orange: ruddy turnstone *Arenaria interpres*), (G) median date for nest initiation of long-tailed skua (*Stercorarius longicaudus*). Note that in 1999 and 2018, one late-emerging *Dryas* plot failed to reach even 50% flowering. No ruddy turnstones were observed to nest in 2018, and no long-tailed skuas to nest in 2014, 2015, and 2018. (The arthropod samples from 2010 were lost in transport, creating a gap in the time series of panels D and E.) In the cumulative distribution graphs, the jagged lines show the number of years (out of 23, except in cases with missing values) in which the respective x-value is exceeded. The dotted lines indicate the 2018 values, with the years to the right of it being the number of years showing a phenology later than 2018. Note that some monitoring plots are consistently late and others early, causing them to reach their maximum value at different points. Panel 2G is missing a dotted line, because in 2018, no long-tailed skuas bred in the area (see Fig 3F). Additional information about data and analyses can be found in S1 Data collection and analyses. Data presented here are available at https://doi.org/10.5281/zenodo.3344483.

For more than two decades, we have documented abundance, reproduction, and phenology across multiple taxa and trophic levels [14,15] in the High Arctic ecosystem at Zackenberg [12]. Yet, based on past observations, we were unable to predict what we encountered in 2018, when unprecedentedly large amounts of snow (Fig 1) resulted in an exceptionally late start of the snow-free season (Fig 2A). Although reproductive failures at the level of populations and species in the Arctic have been reported before [16–18], we now saw a pervasive failure across almost the entire food web.

**Fig 3:**
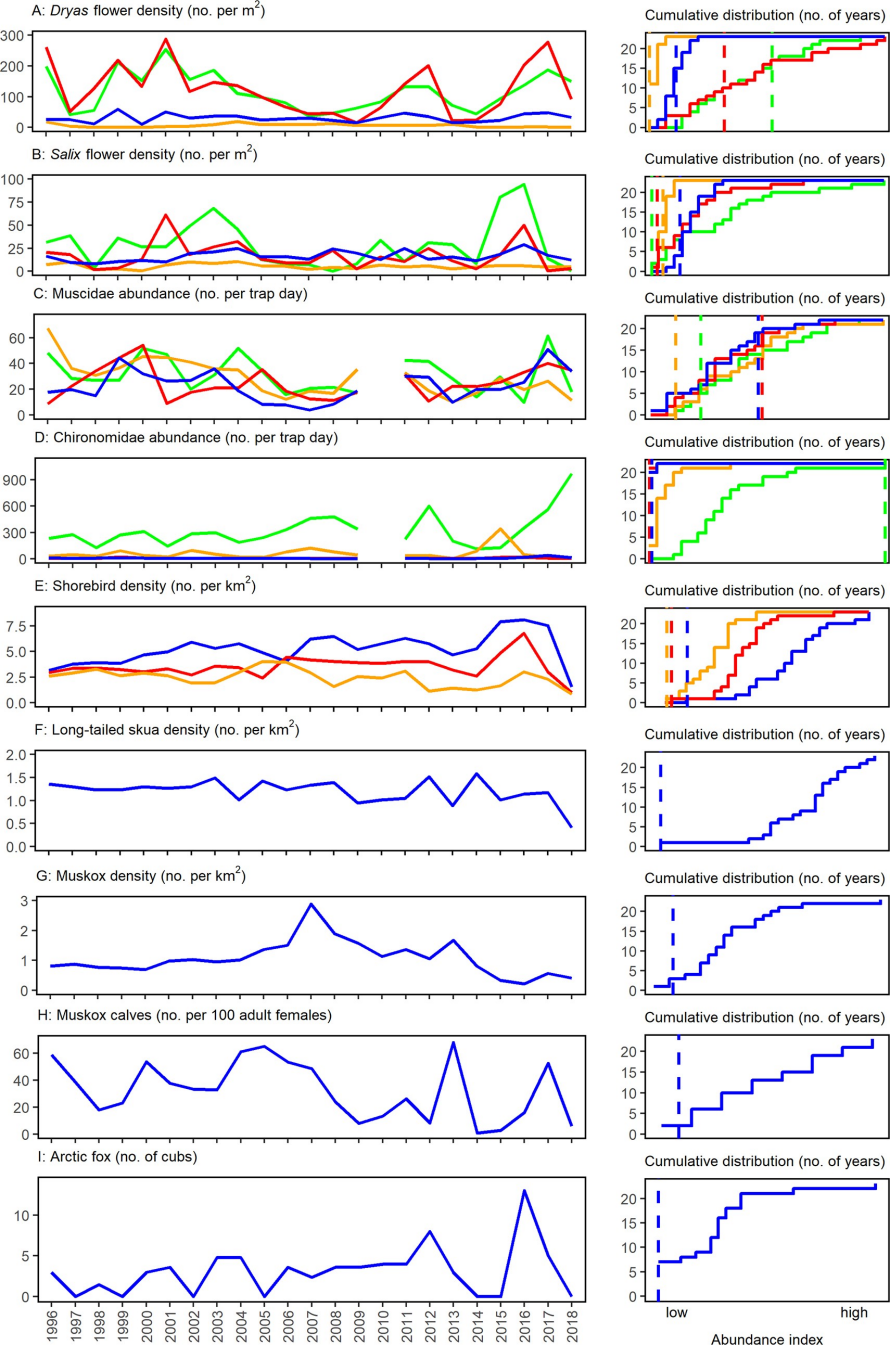
Ecological time series on abundance from Zackenberg in Northeast Greenland. Individual panels show records on abundances and their associated cumulative distribution plots: (A) Flower densities of *Dryas* sp. in four permanent plots, (B) flower densities of *Salix arctica* in four permanent plots, (C) abundances of flies in family Muscidae at four permanent trapping stations, (D) abundances of nonbiting midges in family Chironomidae at four permanent trapping stations, (E) breeding densities of the three most common shorebird species of Zackenberg (blue: Dunlin *Calidris alpina*; red: Sanderling *C*. *alba*; orange: ruddy turnstone *Arenaria interpres*), (F) breeding density of long-tailed skuas, (G) density of muskoxen (*Ovibos moschatus*) in the valley, (H) muskox calves recruitment in the valley, (I) number of Arctic fox (*Vulpes lagopus*) cubs weaned in the valley. (The arthropod samples from 2010 were lost in transport, creating a gap in the time series of panels C and D.) In the cumulative distribution graphs, the jagged lines show the number of years (out of 23, except in cases with missing values) in which the respective x-value is exceeded. The dotted lines indicate the 2018 values, with the years to the left of it being the number of years showing densities lower than 2018. Note that some monitoring plots are characterized by consistently higher densities than others, causing them to reach their maximum value at different points. Additional information about data and analyses can be found in S1 Data collection and analyses. Data presented here are available at https://doi.org/10.5281/zenodo.3344483.

Towards the end of July, when plant growth and animal reproduction usually peak, around 45% of the landscape was still covered in snow. This made it difficult for plants to grow and for animals to access resources. The result was an almost complete reproductive failure of plants and animals of all sizes, as evidenced by patterns in phenology (Fig 2) and abundance (Fig 3). Phenological events across all taxa and trophic levels were dramatically delayed (Fig 2): the onset of flowering and the emergence of arthropods were markedly later than in previous years, and in most cases, the latest observed to date (Fig 2B–2E). Flowering in general occurred so late in the season that seeds were unlikely to develop before the frost set in. The abundance of flowers and arthropods, on the other hand, appeared less affected by the extreme snow conditions: once insects and flowers occurred, they did so at “normal” levels (Fig 3A–3D). For larger animals, abundances were low: only a small fraction of the migratory shorebirds (Fig 3E) and the predatory long-tailed skua (*Stercorarius longicaudus*; Fig 3F) occupied territories compared with previous years. Moreover, because of the late onset of nest initiation (Fig 2F), the very few shorebird eggs that hatched only did so very late in the season. Thus, the young most likely did not have sufficient time and resources to survive until fledging and to prepare for the southward migration. Among the mammals, no Arctic fox (*Vulpes lagopus*) cubs (Fig 3I) and almost no muskox (*Ovibos moschatus*) calves were observed (Fig 3H). Also adult muskox numbers were low (Fig 3G), but the low numbers did not appear specifically linked to the 2018 conditions. In itself, reproductive failure among the mammal species and the long-tailed skua in the area is not uncommon (Figs 2 and 3)—what is uncommon is the extent of the effects across the ecosystem.

In the highly interconnected food webs of the Arctic [14], impacts on one trophic level may easily cascade onto others. For instance, Arctic-breeding shorebirds rely on prey found locally for producing eggs [19]. In 2018, Sanderling (*Calidris alba*) body stores were severely depleted because of the late emergence of their arthropod prey at Zackenberg. Thus, the mean (±SE) body mass of Sanderlings in 2018 (*n* = 31) was 43.9 ± 0.9 g compared with 57.5 ± 0.4 g (*n* = 122) in 2007 to 2017; a significant (ANOVA: F_1,151_ = 248.12, *P* < 0.001) reduction by 24%. The severity of the 2018 conditions were evidenced not only by the near-complete lack of breeding amongst shorebirds but also by five shorebirds found starved to death in 2018—a phenomenon never encountered before. Confronted with a snow-clad landscape and few prey, most migratory birds did not initiate reproduction. Such intertrophic impacts may even extend across years, and the effects of changes in the food base of for instance arthropods (i.e., plants) or shorebirds (i.e., arthropods) in one year may carry over into the following breeding season [14,20,21].

## Implications for the ecosystem

Arctic plants and animals are well adapted to life under extreme climatic conditions [22], and their longevity and temporally dispersed reproductive bouts [23] enable them to cope with the large variability in environmental conditions, both within seasons and between years. Therefore, one nonbreeding year like the one observed in 2018 is hardly devastating for High Arctic species. The worrying perspective here is that the 2018 conditions may offer a peep into the future: Climate change has already resulted in a variety of species and ecosystem-level responses of arctic organisms [11,14]. With less sea ice in the Arctic, we can expect more and more variable amounts of snow in the future [4,24]. It is now well established that climate change includes increased variance in climatic conditions [4]. As a consequence, more extreme events like the 2018 situation in Northeast Greenland may soon be occurring more often than before.

Some extreme events are unique, as for instance, the circumarctic reproductive collapse among shorebirds reported following a volcano eruption in 1992 [16]. Being unlinked to climate change, volcano eruptions will hardly increase in frequency in a changing Arctic. What may increase is the occurrence of extreme snow years like 2018, of rain-on-snow events [25,26], and of episodic snow melt events [27]. A higher incidence of such events may be a game changer for the population dynamics within High Arctic ecosystems, because frequent years with reproductive failure is an issue far more serious than a single event [18,28]. In particular, the consequences are likely to be aggravated among shorter-lived species [22] whose population dynamics is predominantly determined by reproduction [e.g. 29]. However, many arctic plant species are perennials and may reproduce over many—sometimes very many [30]—seasons. Similarly, to the extent that their life cycle is known, a majority of arctic arthropod species has a multiyear life cycle, or emerge from diapause over multiple years, thus reducing the risk of negative population impacts [31]. The overall impacts of extreme events are therefore critically dependent on their frequency and on the conditions in between them.

Importantly, frequent extreme events may not only pose a threat to arctic ecosystems as we know them today but may also contribute to stabilizing population dynamics [32] and to preserving the status quo of Artic communities: With increasing temperatures, more low-latitude species are expected to move north [11], but such species may be less tolerant to the extreme conditions encountered in the Arctic. Extreme events may thus contribute to prevent them from establishing populations there, a suggestion that we should seek to monitor.

## Implications for arctic research

In combination with climate modelling, our unique long-term monitoring data from Zackenberg allow us to evaluate the rareness of the snow conditions in year 2018, to quantify their ecological consequences across multiple taxa and trophic levels, and to detect changes in their frequency over time. Coherent, continuous monitoring of the arctic ecosystems is thus a prerequisite for our ability to actually characterize rare or extreme events and for understanding the likely ecological consequences of such events in a future arctic climate. Our findings from 2018 have brought forth three insights: First, we need to invest equal interest in the variance as in the mean, and this concerns both abiotic and biotic parameters [33]. Second, we should supplement our interest in shifts in temperature with an added focus on shifts in precipitation. Finally, we should step up our efforts to document the dynamics of the High Arctic ecosystem, keeping in mind that sudden shifts in the state of the ecosystem are a possibility. Although our observations from a single High Arctic ecosystem may be considered scanty, they are by far the best ecosystem data available from the region. They suggest that extreme events like the one of 2018 will affect all parts of the arctic ecosystems.

## Supporting information

S1 Data Additional information about data collection and analyses. (DOCX)
