# Supplementary material for: An ecosystem-wide reproductive failure with more snow in the Arctic
Source: PLoS Biol. 2019 Oct 15;17(10):e3000392. doi: 10.1371/journal.pbio.3000392 (PMC6793841; doi:10.1371/journal.pbio.3000392)
Supplement: S1 Data — (DOCX) [file pbio.3000392.s001.docx]

Supporting Information for

**An ecosystem-wide reproductive failure with more snow in the Arctic**

Niels Martin Schmidt, Jeroen Reneerkens, Jens Hesselbjerg Christensen, Martin Olesen and Tomas Roslin

**Precipitation data and analyses**

Snowfall data is not readily available from many sites across the Arctic region, including Greenland. Hence, we used high resolution models from regional climate models at a horizontal resolution of 5x5km. For the Pan-Arctic perspective we based our analysis on ERA5-reanalysis data available from the Copernicus Climate Change Service [1]. Similarly, for the Greenland scale, we based our analysis on data from the regional climate model HIRHAM5 [2,3] driven with ERA-Interim reanalysis data [4] on the lateral boundaries for the period 1980 to 2014. From 2014 to 2017 ERA-interim has been downscaled with HIRLAM [5] and from 2017 to 2018 with HARMONIE [6]. See also [www.polarportal.org](http://www.polarportal.org) where this information is made available in an operational context.

To extent the time series on snow precipitation back in time, we reconstructed the accumulated snowfall averaged over the drainage basin 3.1 defined by Zwally et al. [7], representing the Greenland Ice Sheet drainage basin closest to Zackenberg.

Prior to analysis, time series were standardized to zero mean and a standard deviation of one. Hence, data presented show pixel-level or annual deviation of the 2018 season from the standardized long-term mean.

**Ecological data and analyses**

All data on biota collected at Zackenberg have followed the same field protocol since the implementation of the BioBasis program in 1996 [8].

***Plants***

Data on *Dryas* sp. and *Salix arctica* flowering phenology and abundance were collected in designated, permanent monitoring plots. The number of permanent plots has been adjusted over the years (ref manual), and we therefore only present flowering data from plots that has been monitored during the entire study period 1996-2018 (DRY1-4, SAL1-4). In each plot, the number of buds, flowers (or catkins) and senescent flowers (or catkins) are counted weekly from snow-melt until early autumn. From these data we calculated the day-of-year of 50% flowering by linear interpolation between the weekly estimates for each plot separately [9]. Abundance of flowers were estimated once a year at the peak of flowering by counting all flowers within each plot separately [8].

***Arthropods***

Data on arthropod emergence phenology and abundance were collected at four permanent trapping stations. Three of the trapping stations consisted of 4-8 yellow pitfall traps (ART2, ART3 and ART5), while one trapping station had two window traps (ART1) [8]. More trapping stations are or have been operated at Zackenberg, but not consistently so over the entire study period and were hence not included in the study. The four trapping stations are located in the dominant vegetation types of the area (ART1 and ART2: fen and grassland-like vegetation; ART3: *Cassiope* heath and ART5: *Dryas* heath), also corresponding to a gradient in timing of snow-melt [10]. Individual traps were emptied once a week and sorted to family-level. We expressed catches as numbers per trap day as these may vary between individual traps. We only examined data collected in the months June through August, since all trapping stations have been consistently monitored in these three months during the entire study period. From the weekly data, we calculated the day-of-year of 50% emergence by linear interpolation between the weekly estimates for each trapping station separately [9]. Similarly, we calculated the total number of catches per trap day per trap stations in the months June through August.

The arthropod community at Zackenberg consists of numerous taxa [11], and for simplicity we only present data on the two most dominant arthropod families, Chironomidae and Muscidae [9], of which Muscidae are also the functionally dominant pollinators in the region [12]. Note that Muscidae includes Anthomyiidae, and Chironomidae include Ceratopogonidae [9].

***Birds***

Data on the avian community at Zackenberg were collected within a permanent, designated 15.8 km^2^ bird census area [8]. Breeding abundances of shorebirds and Long-tailed skua were estimated each year in mid- to late June (depending on snow conditions), where the census area was surveyed on foot following the same, predefined route. Within the census area, all territories of breeding birds are mapped using observations of bird behaviour: Birds in pairs as well as singing and otherwise vocalising birds were regarded as representing a territory [13]. The survey was usually completed within five days, and with similar effort every year, and no censuses were conducted on days with inclement weather. Observations of nests and broods throughout the season were used to validate the initial census whenever possible. When the number of breeding pairs/territories was given as an interval between certain and possible territories, we used the median as our estimate of the number of breeding pairs.

Avian breeding phenology (nest initiation date) was estimated by floating of eggs found in nests [14] for each of the three most abundant shorebird species (Dunlin, Sanderling and Ruddy turnstone) and for the Long-tailed skua. For each species, we expressed their breeding phenology as the median date of nest initiation [15].

***Sanderling body weights***

Adult Sanderlings were captured on their nests at Zackenberg using small clapnets annually between 2007-2017 [16–18]. In 2018, we did not catch the Sanderling on the single clutch found that summer. Instead, we were able, that season only, to catch non-breeding Sanderlings foraging on kitchen waste from the Zackenberg field station using the same clapnets. Each bird caught was weighed to the nearest 0.1 g using electronic scales. Sanderlings increase in body mass from the start of incubation until hatch [19], also in our study [Reneerkens J unpubl. obs.]. Therefore, to best compare Sanderling weights in 2018 with those of an incubating individual, we only included in our analysis body weights of Sanderlings weighed during their first week of incubation. The age of a clutch was determined by placing two of the usually four eggs in warm water upon discovery of the clutch and using their degree of flotation following [14] assuming an incubation duration of 22 days [16].

***Mammals***

Data on muskox density and calf recruitment were obtained in a designated 47 km^2^ muskox census area [8]. Since the implementation in 1996, the muskoxen within the census area has been registered once a week during July and August. Additional censuses have been conducted but not consistently so across the entire study period [20].

The number of weaned Arctic foxes cub was estimated from multiple visits to known dens in the Zackenberg valley in late July [21]. Not all dens were known from the beginning of the monitoring at Zackenberg [8] and the number of fox cubs was therefore weighted by the fraction of dens surveyed [21].

**References**

1. ERA5 CCCSC (2017) ERA5: Fifth generation of ECMWF atmospheric reanalyses of the global climate. Copernicus Climate Change Service Climate Data Store (CDS). <https://cds.climate.copernicus.eu/cdsapp#!/home> (accessed 2019-05-08)

2. Langen PL, Mottram RH, Christensen JH, Boberg F, Rodehacke CB, Stendel M, van As D, Ahlstrøm AP, Mortensen J, Rysgaard S, Petersen D, Svendsen KH, Aoalgeirsdóttir G, Cappelen J (2015) Quantifying Energy and Mass Fluxes Controlling Godthåbsfjord Freshwater Input in a 5-km Simulation (1991-2012). J Climate 28: 3694-3713.

3. Christensen OB, Drews M, Christensen JH, Dethloff K, Ketelsen K, Hebestadt I, Rinke A (2006) The HIRHAM regional climate model version 5. DMI Technical Report No. 06-17.

4. Dee DP, Uppala SM, Simmons AJ, Berrisford P, Poli P, Kobayashi S, Andrae U, Balmaseda MA, Balsamo G, Bauer P, Bechtold P, Beljaars ACM, van de Berg L, Bidlot J, Bormann N, Delsol C, Dragani R, Fuentes M, Geer AJ, Haimberger L, Healy SB, Hersbach H, Hólm EV, Isaksen L, Kållberg P, Köhler M, Matricardi M, McNally AP, Monge-Sanz BM, Morcrette JJ, Park BK, Peubey C, de Rosnay P, Tavolato C, Thépaut JN, Vitart F (2011) The ERA-Interim reanalysis: configuration and performance of the data assimilation system. Q J R Meteorol Soc 137: 553-597.

5. Eerola K (2006) About the performance of HIRLAM version 7.0. HIRLAM Newsletter 51: 93-102.

6. Bengtsson L, Andrae U, Aspelien T, Batrak Y, Calvo J, de Rooy W, Gleeson E, Hansen-Sass B, Homleid M, Hortal M, Ivarsson KI, Lenderink G, Niemelä S, Nielsen KP, Onvlee J, Rontu L, Samuelsson P, Munoz DS, Subias A, Tijm S, Toll V, Yang X, Køltzow MØ (2017) The HARMONIE-AROME Model Configuration in the ALADIN-HIRLAM NWP System. Mon Wea Rev 145: 1919-1935.

7. Zwally HJ, Mario BG, Matthew AB, Jack LS (2012) Antarctic and Greenland Drainage Systems, GSFC Cryospheric Sciences Laboratory. <http://icesat4.gsfc.nasa.gov/cryo_data/ant_grn_drainage_systems.php> (accessed 2018-06-02).

8. Schmidt, N. M., Hansen, Lars H., Hansen, Jannik, Berg, T. B., and Meltofte, H. (2018) BioBasis - conceptual design and sampling procedures of the biological programme of Zackenberg Basic, 21^st^ edition. Roskilde: Department of Bioscience, University of Aarhus, Denmark. 109 p.

9. Schmidt NM, Mosbacher JB, Nielsen PS, Rasmussen C, Høye TT, Roslin T (2016) An ecological function in crisis? - the temporal overlap between plant flowering and pollinator function shrinks as the Arctic warms. Ecography 39: 1250-1252.

10. Bowden JJ, Hansen OLP, Olsen K, Schmidt NM, Høye TT (2018) Drivers of inter-annual variation and long-term change in High-Arctic spider species abundances. Polar Biol ??.

11. Wirta HK, Vesterinen EJ, Hambäck PA, Weingartner E, Rasmussen C, Reneerkens J, Schmidt NM, Gilg O, Roslin T (2015) Exposing the structure of an Arctic food web. Ecol Evol 5: 3842-3856.

12. Tiusanen M, Herbert PDN, Schmidt NM, Roslin T (2016) One fly to rule them all - muscid flies are the key pollinators in the Arctic. Proc R Soc B 283: 20161271.

13. Meltofte H (2006) Wader populations at Zackenberg, high-arctic Northeast Greenland, 1996-2005. Dansk Orn Foren Tidsskr 100: 16-28.

14. Hansen J, Schmidt NM, Reneerkens J (2011) Egg hatchability in high Arctic breeding wader species Charadriiformes is not affected by determining incubation stage using the egg flotation technique. Bird Study 58: 522-525.

15. Meltofte H, Høye TT, Schmidt NM, Forchhammer MC (2007) Differences in food abundance cause inter-annual variation in the breeding phenology of High Arctic waders. Polar Biol 30: 601-606.

16. Reneerkens J, Grond K, Schekkerman H, Tulp I, Piersma T (2011) Do uniparental sanderlings Calidris alba increase egg heat input to compensate for low nest attentiveness? Plos One 6: e16834.

17. Reneerkens J, van Veelen P, van der Velde M, Luttikhuizen P, Piersma T (2014) Within-population variation in mating system and parental care patterns in the Sanderling (Calidris alba) in northeast Greenland. Auk Ornithol Adv 131: 235-247.

18. Reneerkens J, Schmidt NM, Gilg O, Hansen J, Hansen LH, Moreau J, Piersma T (2016) Effects of food abundance and early clutch predation on reproductive timing in a high Arctic shorebird exposed to advancements in arthropod abundance. Ecol Evol 6: 7375-7386.

19. Soloviev MY, Tomkovich PS (1997) Body mass changes in waders (Charadrii) in a high arctic area at northern Taimyr, Siberia. Journal f++r Ornithologie 138: 271-281.

20. Schmidt NM, Pedersen SH, Mosbacher JB, Hansen LH (2015) Long-term patterns of muskox (*Ovibos moschatus*) demographics in high arctic Greenland. Polar Biol 38: 1667-1675.

21. Schmidt NM, Ims RA, Høye TT, Gilg O, Hansen LH, Hansen J, Lund M, Fuglei E, Forchhammer MC, Sittler B (2012) Response of an arctic predator guild to collapsing lemming cycles. Proc R Soc B 279: 4417-4422.
